# Assessment of food safety knowledge, attitudes and practices of street food vendors in Chattogram city, Bangladesh: A cross‐sectional study

**DOI:** 10.1002/puh2.16

**Published:** 2022-07-29

**Authors:** Mohammad Tazrian Abid, Md. Hasan Al Banna, Mohammad Hamiduzzaman, Abdul‐Aziz Seidu, Satyajit Kundu, Humayra Rezyona, Tasnim Rahman Disu, Nargees Akter, Md Khaleduzzaman, Bright Opoku Ahinkorah, Md Shafiqul Islam Khan

**Affiliations:** ^1^ Faculty of Nutrition and Food Science Patuakhali Science and Technology University Patuakhali Bangladesh; ^2^ Department of Food Microbiology Faculty of Nutrition and Food Science Patuakhali Science and Technology University Patuakhali Bangladesh; ^3^ Faculty of Health Southern Cross University, Gold Coast Queensland Australia; ^4^ Department of Real Estate Management Takoradi Technical University Takoradi Ghana; ^5^ Centre for Gender and Advocacy Takoradi Technical University Takoradi Ghana; ^6^ College of Public Health Medical and Veterinary Sciences James Cook University Townsville Australia; ^7^ Department of Biochemistry and Food Analysis Patuakhali Science and Technology University Patuakhali Bangladesh; ^8^ Department of Food and Nutrition College of Home Economics, Azimpur Dhaka Bangladesh; ^9^ Institute of Public Health Nutrition, Mohakhali Dhaka Bangladesh; ^10^ Department of Geography and Environmental Studies University of Chittagong Chittagong Bangladesh; ^11^ School of Public Health Faculty of Health University of Technology Sydney Sydney Australia

**Keywords:** attitudes, Bangladesh, food safety, knowledge, practices, street food vendors

## Abstract

**Introduction:**

Street food has become popular in developing countries due to its affordability, availability and taste. Maintaining the quality and safety of street food is linked to the vendors’ appropriate food handling practices to reduce foodborne illness. Therefore, this study aimed to assess food safety knowledge, attitudes and practices of street food vendors in Chattogram city, Bangladesh.

**Methods:**

A cross‐sectional study was carried out among 302 street food vendors from December 2020 to March 2021. Data were collected by in‐person interviews through a structured questionnaire. Independent sample *t*‐tests and one‐way analysis of variance (ANOVA) were used to compare food safety knowledge, attitudes, and practices scores across socio‐demographic variables.

**Results:**

The mean score of food safety knowledge, attitudes and practices was 8.99 (SD = 4.17, range: 1–18), 8.46 (SD = 3.51, range: 1–16) and 17.78 (SD = 5.74, range: 1–34), respectively. The food safety knowledge scores significantly (*p* < 0.05) differed by the participants’ age, marital status, income, residence and work experience. The average food safety attitudes score significantly (*p* < 0.05) varied by age, marital status, income, and education level. The average food safety practices score significantly (*p* < 0.05) differed by the respondents’ education level and work experience.

**Conclusion:**

Our findings suggest that food safety knowledge, attitudes, and practices were poor among street food vendors. Therefore, there is a need for strategies and intervention programs such as food safety training and awareness campaigns as well as financial support to improve food safety knowledge, attitudes, and practices which help to reduce foodborne illness.

## Graphical abstract

Street food vendors’ appropriate knowledge, attitudes, and practices regarding food safety are vital for maintaining the quality and safety of street food; therefore, this study assessed food safety knowledge, attitudes and practices of street food vendors in Chattogram city, Bangladesh, and found poor knowledge, attitudes and practices regarding food safety. Several sociodemographic factors are associated with street food vendors’ food safety knowledge, attitudes and practices. These findings highlight a need for strategies and intervention programs to improve food safety knowledge, attitudes and practices which help to reduce foodborne illness.

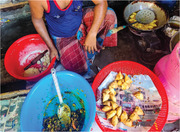

## INTRODUCTION

Street‐vended food (SVF) includes ready‐to‐eat foods and a variety of beverages often sold in stations, markets, parks and zones centralized in education institutions and industrial areas [1, 2]. Street food has become a part of diet culture, especially in urban areas due to its affordability, taste and availability [3]. Moreover, people often have to rely on street foods due to their hectic lifestyles such as they are too busy to spend time cooking at home [4]. In low‐and middle‐income countries, street food businesses contribute to the economy which helps the low‐income peoples’ livelihood [5]. However, in these countries including Bangladesh, street food vending systems are beyond the control, regulation and supervision of the respective authorities [6]. Therefore, the quality and safety of street food, especially with the knowledge of foodborne illnesses and diseases among street food vendors, remains a matter of concern.

A lack of education and unawareness about the hygiene of vending sites and appropriate handling of foods is common among street food vendors [7, 8]. The vendors’ inadequate knowledge of microbiological hazards (e.g., provision of safe water, diversity and mobility) of street food, coupled with poor infrastructure and facilities creates difficulty in safe vending operations [9, 10]. Additionally, poor personal hygiene practices, lack of sanitary measures at or surrounding the vending site, and unhygienic serving utensils (i.e., paper, plastic plate, spoon, etc.) as well as untreated water used by street food vendors can all act in the transmission of pathogenic microorganisms via foods to humans [11]. Evidence from developing countries reported that SVF has been associated with outbreaks of foodborne diseases [8, 12]. Highly pathogenic coliform bacteria have been found in SVF in several countries including Bangladesh [13], and the consumption of street food is suspected to facilitate the spread of pathogens resistant to antimicrobials [14].

In Bangladesh, approximately 130 types of SVF are available [15]. One in every six street food consumer in this country become ill after consuming SVFs, and around 30 million people experience foodborne diseases each year [16, 17]. A recent review of Bangladeshi literature on the microbiological safety of SVFs reported that most of the SVF samples from all examined places had a high microbial load, with *E. coli* found to be the predominant pathogen [18]. Several studies related street food vendors’ lack of knowledge and practices with unsafe food and foodborne diseases [19, 20]. Consequently, SVFs are perceived as major public health problems in Bangladesh [5].

Rapid urbanization and unemployment are two major reasons for increasing SVF shops in Bangladesh [5]. Chattogram, the second‐largest city in the country, has an unemployment rate of 4.70%, and most street food vendors choose this informal business for the sake of survival and livelihood [21]. Moreover, as the south‐eastern coastal and tourist area of Bangladesh, Chattogram is highly vulnerable to food safety because of climatic factors which impact the survival of microbial growth via altering the temperature in addition to changing the water quality and availability.

The knowledge, attitudes and practices (KAP) model is widely used to explore food safety issues at different sectors of the food production or food value chain. Acquiring appropriate knowledge regarding food safety is essential because it can potentially reduce the incidence of foodborne diseases [22]. Food handlers’ level of food safety knowledge may positively impact on their attitudes, which are subsequently converted into practices [23]. Food handlers’ attitude is an important factor that could influence food safety behavior and practices [24, 25]. Banna et al. [26] showed that higher food safety knowledge is associated with a good level of food safety practices among food handlers. Previous studies reported that the level of awareness, attitude and food safety training were significantly related to the street food vendors’ hygienic practices [27, 28, 29]. However, despite having good food safety knowledge among street food vendors, their attitudes and practices might be low [19]. Though few previous studies on street food vendors’ knowledge, attitudes and practices have been conducted in different cities, for example, Jashore and Dhaka in Bangladesh [19, 30], to our knowledge, no studies have been conducted in Chattogram.

An early exploration of food safety knowledge, attitudes and practices among street food vendors in Chattogram city is important to avoid foodborne disease outbreaks. The current research will fill the gaps of literature and provide baseline data on food safety knowledge, attitudes and practices among street food vendors in this area. Thus, the findings of this study may set a benchmark for the local authorities and government to enforce sanitary conditions and food safety regulations in the region. Therefore, the purpose of this study was to assess the knowledge, attitudes and practices regarding food safety among a sample of street food vendors in Chattogram, Bangladesh.

## MATERIALS AND METHODS

### Study design, subjects, and sampling

A cross‐sectional study, involving a field survey, was conducted among 302 street food vendors to explore the food safety knowledge, attitudes and practices. A sample size of 384 was determined using Cochran's formula [31] by assuming a 50% prevalence of expected food safety knowledge, attitudes and practices among street food vendors in Chattogram city, Bangladesh (since no previous study of this type in the study area), a 95% confidence interval (CI) and 5% margin of error. Later, a modified Cochran's formula [31] was used for calculating the adjusted sample size in a small population (assuming 1000 street food vendors work in this study area), giving a minimum sample size of 277.

A systematic random sampling technique was employed to include the study subjects. Initially, data collectors visited conveniently possible places (such as playgrounds, parks, bus terminals, and markets) where street food might sell and first street food shop was randomly selected and then next street food shop was selected after 3 shops using a sampling interval (*k* = N/n), found 341 street food shops for recruiting the study subjects. Subsequently, data collectors enrolled 320 street food vendors using the lottery method from the identified shops. Data collectors assigned a unique identifying number for each vendor in a particular street food shop, these numbers were written on a piece of paper, folded and placed in a box with other pieces of folded paper with vendors’ unique numbers. The data collector drew a number from the box and assessed the individual for the study. This study applied the following inclusion criteria: (i) who sold ready‐to‐eat foods which was fully or partially homemade, (ii) who worked as permanent staff, and (iii) only adults (age ≥18 years). Permanent staff worked in this street food selling profession in long‐term for their livelihood, therefore, evaluating their food safety knowledge, attitudes and practices are more important to reduce foodborne illness than temporary workers who may switch their occupation. Eighteen street food vendors declined to participate due to various reasons including busy for business (*n* = 8), lack of interest in participating (*n* = 6) and unable to understand the value of research (*n* = 4), and withdrawn their participation. Finally, a total of 302 street food vendors were included in this study.

### Study setting

The south‐eastern region of Bangladesh – Chattogram city – was chosen as the empirical setting of this study for four reasons. Firstly, being a coastal area and financial centre of Bangladesh, Chattogram city has a high population density (total population: 8.6 million) and poverty, where a large number of low‐wage earners including manufacturing and transport workers and school children heavily rely on street foods [32]. Secondly, street food partially meets the food and nutritional requirements of low‐and middle‐income residents of Chattogram at affordable prices, therefore becoming an integral part of the community. Thirdly, a growing body of research conducted in Dhaka, the capital of Bangladesh, identified about eight million (55%) of its residents consume some street food every day and 300,000 people are engaged in selling street food [5]. This study [5] also reported poor hygiene practices among the vendors that helped to explain the epidemiology of foodborne symptoms and diseases in the residents of Bangladesh's capital city, whereas no studies were found on food safety and hygiene practices of street vendors in Chattogram. Finally, food safety, especially the safety of SVF remains a major public health concern in this city [33, 34], making this area suitable for the research.

### Data collection

Written consent (verbal consent in case of illiterate respondents) was obtained from each participant and he/she was informed about the purpose of the study, associated risks, and maintaining anonymity and confidentiality in reporting the study findings.

Six trained data collectors (two from the study authors and rest of them hired as voluntary data collectors) obtained data by in‐person interviews over four months (from December 2020 to March 2021) through a pre‐tested structured questionnaire. An online training session was arranged by the principal investigator and first author of this study to train data collectors about different parts of the questionnaire, data collection methods, and inclusion/exclusion criteria of the study. The English version of the questionnaire was retrieved from the studies of Hossen et al.; Ma et al. and Samapundo et al. [19, 35, 36], and translated into the native language (Bangla) for better communication between data collectors and respondents. Prior to the final version of the questionnaire being administered to study participants, a pre‐test of the questionnaire was done among a randomly selected small group of street food vendors (*n* = 10). Based on the pre‐tested results of the questionnaire, some modifications were made to the different sections of the questionnaire where the questions were unclear. For example, we removed the item “Influenza can be transmitted by aerosols rather than food” from food safety knowledge section. The piloting samples were not included in the final analysis. The Cronbach's alpha of different sections of the questionnaire ranges from 0.72 to 0.81, which indicates an acceptable internal consistency. Each interview took 20–25 min to accomplish. Unique identifier codes were assigned to the questionnaire to assure the anonymity of the participants.

### Outcomes measures

A 51‐item vendor's food safety knowledge, attitude and practice scale was used. This scale included 18 questions on food safety knowledge (i.e., personal hygiene, cleaning, cross‐contamination, foodborne disease and pathogens). All questions had three possible answers: “true”, “false” and “don't know”. If the answer to a question was “true”, it was given a score of 1, and if the answer was “false” and or “don't know”, it was given a score of 0. Each participant could receive a total score between 0 and 18 on the vendor's food safety knowledge section. Bloom's cut‐off point (good: 80%–100%; moderate: 50%–79%; and poor: <50%) was employed to categorize the participants’ overall food safety knowledge [37].

A 16‐item food safety attitude section was also included in the scale, with three possible answers (“agree”, “disagree” and “no idea”). This section included information on personal hygiene, food storage, preservation, cross‐contamination and cleaning. For each response of “agree”, 1 point was denoted, whereas for responses of “disagree” and “no idea” the score was 0. A maximum of 16 points (0–16) could be allocated to the food safety attitude section. Bloom's cut‐off point (positive: 80%–100%; neutral: 60%–79%; and negative: <60%) was used to categorize the vendor's attitudes toward food safety [37].

The questionnaire also included a section of 17‐item to examine the vendors’ hygiene practices, with three possible answers (“always”, “sometimes” and “never”). For “always” and “sometimes” responses, 2 and 1 points were given, respectively, otherwise 0 points were given. The total score on the vendors’ hygiene practices section ranged from 0 to 34. To determine hygiene practices levels as good, moderate and poor we used Bloom's cut‐off point in the same way as assessing vendor's food safety knowledge levels.

After conducting the interview, the research staff observed the environment and sanitary conditions (such as whether any flies around the stall, having waste disposal and potable water facilities, cleanliness, etc.) around the stall by using a 9‐item checklist to determine the environmental parameters of vending sites. For example, each checklist had two options (yes vs. no options) for marking the items such as “No flies around the stall”, “stall is far from rubbish” etc.

### Explanatory variables

The respondents’ socio‐demographic characteristics that is, gender, age, work experience, educational level, marital status, monthly income, residence and food safety training were used as explanatory variables.

### Ethics

All study protocols and procedures were reviewed and approved by the department of the principal author (approval number: FMB:15/12/2020:05). Written consent was obtained from the participants (street food vendors) after discussing the purpose of the study, confidentiality of their data, and after assuring the participant that this research would not be harmful to them.

### Data analyses

Data were analyzed by Statistical Package for the Social Sciences (SPSS) software (IBM version 23.0, Armonk, NY, USA). Descriptive statistics such as frequencies, percentages, means, and standard deviations were computed to check the univariate outliers of the variables. The normality of data was checked by using the Shapiro–Wilk test and the data were found to be normally distributed. Independent sample *t*‐tests and one‐way analysis of variance (ANOVA) were used to compare food safety knowledge, attitudes, and practices scores across socio‐demographic characteristics. Spearman rank correlation was used for the ordinal variables (such as age, education, work experience and household income) and Pearson's correlation was used for continuous variables (food safety knowledge, attitude and practice score) to produce bivariable level statistics. *P*‐values were statistically significant at <0.05 (95% confidence interval).

## RESULTS

The participants’ characteristics are presented in Table 1. The response rate of the study participants was 94.4%. Of the 302 participants, the majority were male (77.5%), 37.1% had no formal education, and 96.7% did not have food safety training. The mean age was 34.85 years (SD: ±10.58 years), where 75.2% were 25–50 years old.

**TABLE 1:** Sociodemographic characteristics of study participants (*n* = 302)

Characteristics	Frequency	Percentage
Gender		
Male	234	77.5
Female	68	22.5
Age (years)		
Mean (SD): 34.85 (10.58)		
<25	49	16.2
25–50	227	75.2
>50	26	8.6
Experience of work (years)		
<5	78	25.8
5–9	153	50.7
10–20	62	20.5
>20	9	3.0
Educational level		
No formal education	112	37.1
Primary	123	40.7
Secondary	55	18.2
Higher secondary	7	2.3
Under graduation	5	1.7
Marital status		
Single	115	38.1
Married	165	54.6
Divorced, separated or widowed	22	7.3
Monthly Income (BDT)^a^		
<5000	19	6.3
5000–10000	105	34.8
10001–20000	92	30.5
>20000	86	28.5
Residence		
City area	194	64.2
Sur urban	57	18.9
Rural area	51	16.9
Food safety training		
Yes	10	3.3
No	292	96.7

^a^ BDT, Bangladeshi Taka (1 USD = 85.65 BDT, Oct 30, 2021, 11:11 AM UTC).

Table 2 summarizes the participants’ food safety knowledge. Low knowledge of foodborne pathogens was found in participants, for example, less than one‐fourth reported knowing *Salmonella* (23.8%) and *Staphylococcus* (20.9%) are foodborne pathogens. However, participants reported a higher knowledge about foodborne diseases, around two‐thirds correctly answered that bloody diarrhea (67.5%) and typhoid (62.3%) can be transmitted by contaminated food. About 52.6% either did not know or thought it was necessary to take leave from work during infectious skin disease. The majority of participants (73%) were unaware that children, pregnant women and older adults were at equal risk of contracting foodborne diseases, but 71.5% and 68.2% of participants were knowledgeable about washing hands and gloves use, respectively. According to Bloom's cut‐off point, 39.4% had poor knowledge and 52.3% had moderate level knowledge. The mean score of knowledge was 8.99 (SD = 4.17, range: 1–18). The average food safety knowledge scores significantly (*p* < 0.05) differed by the participants’ age, marital status, income, residence and work experience (Table 5).

**TABLE 2:** Assessment of food safety knowledge of street food vendors in Chattogram city, Bangladesh (*n* = 302)

	Responses, *n* (%)		
Statement	True	False	Don't know
1. Abortion in pregnant women can be induced by foodborne disease.	221 (73.2)	8 (2.6)	73 (24.2)
2. Bloody diarrhea can be transmitted by contaminated food.	204 (67.5)	77 (25.5)	21 (7.0)
3. Swollen cans can contain microorganisms.	162 (53.6)	49 (16.2)	91 (30.1)
4. During infectious disease of the skin, it is necessary to take leave from work.	143 (47.4)	74 (24.5)	85 (28.1)
5. Eating and drinking in the work place increases the risk of food contamination.	197 (65.2)	53 (17.5)	52 (17.2)
6. *Hepatitis A* virus is a foodborne pathogen.	105 (34.8)	116 (38.4)	81 (26.8)
7. Microbes are in the skin, nose and mouth of healthy handlers.	184 (60.9)	51 (16.9)	67(22.2)
8. *Salmonella* is among the foodborne pathogens.	72(23.8)	95 (31.5)	135 (44.7)
9. *Staphylococcus* is among the foodborne pathogens.	63 (20.9)	90 (29.8)	149 (49.3)
10. Typhoid fever can be transmitted by contaminated food.	188 (62.3)	53 (17.5)	61 (20.2)
11. Using gloves while handling food reduces the risk of food contamination.	206 (68.2)	53 (17.5)	43 (14.2)
12. Washing hands before work reduces the risk of food contamination.	216 (71.5)	40 (13.2)	46(15.2)
13. AIDS can be transmitted by contaminated food.	61(20.2)	192 (63.6)	49(16.2)
14. Children, healthy adults, pregnant women and older individuals are at equal risk for food poisoning.	81 (26.8)	177 (58.6)	44 (14.6)
15. Food prepared in advance reduces the risk of food contamination.	207(68.5)	42(13.9)	53(17.5)
16. Proper cleaning and sanitization of utensils decrease the risk of food contamination.	64(21.2)	188 (62.3)	50(16.6)
17. Reheating cooked foods can contribute to food contamination.	183(60.6)	62 (20.5)	57 (18.9)
18. Washing utensils with detergent leaves them free of contamination.	159 (52.6)	67 (22.2)	76 (25.2)
Food safety knowledge score quality, *n* (%)	Poor	Moderate	Good
	119 (39.4)	158 (52.3)	25 (8.3)

*Note*: Mean food safety knowledge score: Mean = 8.99 (SD = 4.17, range = 1–18).

Table 3 represents the findings of vendor's food safety attitude. About 74.5% of participants agreed on storing raw and cooked foods separately, whereas more than two‐thirds agreed that wearing masks (65.9%), gloves (70.9%) and caps (66.2%) is an important practice to reduce the risk of food contamination. A large proportion (71.2%) believed that foodborne diseases can be prevented by hygiene precautions. According to Bloom's cut‐off point, 12.6% had a positive attitude and 62.9% were a negative. The average score for attitude towards food safety was 8.46, (SD = 3.511, range: 1–16). The average attitude score significantly (*p* < 0.05) varied by age, marital status, income and education level (Table 5).

**TABLE 3:** Assessment of food safety attitudes of street food vendors in Chattogram city, Bangladesh (*n* = 302)

	Responses, *n* (%)		
Statements	Agree	Disagree	No idea
1. Proper hand hygiene can prevent foodborne diseases.	215 (71.2)	74 (24.5)	13 (4.3)
2. Raw and cooked foods should be stored separately to reduce the risk of food contamination.	225 (74.5)	59 (19.5)	18 (6.0)
3. It is necessary to check the temperature of refrigerators/freezers periodically to reduce the risk of food contamination.	102 (33.8)	101 (33.4)	99 (32.8)
4. The health status of workers should be evaluated before employment.	117 (38.7)	101 (33.4)	84 (27.8)
5. The best way to thaw chicken a bowl of cold water.	119 (39.4)	127 (42.1)	56 (18.5)
6. Wearing masks is an important practice to reduce the risk of food contamination.	199 (65.9)	71 (23.5)	32 (10.6)
7. Wearing gloves is an important practice to reduce the risk of food contamination.	214 (70.9)	64 (21.2)	24 (7.9)
8. Wearing caps is an important practice to reduce the risk of food contamination.	200 (66.2)	77 (25.5)	25 (8.3)
9. Dish towels can be a source of food contamination.	190 (62.9)	93 (30.8)	19 (6.3)
10. Knives and cutting boards should be properly sanitized to prevent cross contamination.	172 (57.0)	90 (29.8)	40 (13.2)
11. Food handlers who have abrasions or cuts on their hands should not touch food without gloves.	201(66.6)	83 (27.5)	18 (6.0)
12. Well‐cooked foods are free of contamination.	135 (44.7)	128 (42.4)	39 (12.9)
13. Can a closed can/jar of cleaning product be stored together with closed cans and jars of food products.	68 (22.5)	201 (66.6)	33 (10.9)
14. Defrosted foods can be refrozen.	82 (27.2)	170 (56.3)	50 (16.6)
15. The ideal place to store raw meat in the refrigerator is on the bottom shelf.	144 (47.7)	99 (32.8)	59 (19.5)
16. Eggs must be washed after purchase as soon as possible.	173 (57.3)	92 (30.5)	37 (12.3)
Food safety attitudes score quality, *n* (%)	Positive	Neutral	Negative
	38 (12.6)	74 (24.5)	190 (62.9)

*Note*: Mean food safety attitudes score: Mean = 8.46 (SD = 3.511, range = 1–16).

Food safety practices in vendors are illustrated in Table 4. Most participants reported always or sometimes washing their hands before processing foods (84.1%), and before and after touching unwrapped raw foods (76.2%), but 82.8% of participants reported never or sometimes using soaps or detergents to wash their hands. Wearing an apron (79.5%), cap (77.1%), mask (69.9%) and glove (70.8%) were reported to be poorly practiced during work. About 51.7% of participants always reused the oil in cooking. Bloom's cut‐off point stated that only 4.6% presented good hygiene practices. The average score for hygiene practices was 17.78, (SD = 5.74, range: 1–34). As shown in Table 5, the average food safety practices score significantly (*p* < 0.05) varied by the respondents’ education level and experience of work.

**TABLE 4:** Assessment of food safety practices of street food vendors in Chattogram city, Bangladesh (*n* = 302)

	Responses, *n* (%)		
Questions	Always	Sometimes	Never
1. Do you wash your hands before processing food?	121 (40.1)	130 (43.0)	51 (16.9)
2. Do you wash your hands after you touch food?	74 (24.5)	163 (54.0)	65(21.5)
3. Do you wash your hands before and after touching unwrapped raw foods?	93 (30.8)	137 (45.4)	72 (23.8)
4. Do you use soaps/detergents to wash your hands?	52 (17.2)	102 (33.8)	148 (49.0)
5. Do you keep your nails short and remove all adornments before starting activities?	79 (26.2)	159 (52.6)	64 (21.2)
6. Do you handle food at work when you have diarrhea?	81 (26.8)	158 (52.3)	63 (20.9)
7. Do you clean the work area before starting the work?	87 (28.8)	164 (54.3)	51 (16.9)
8. After using the toilet, do you wash your hands with soap or detergent?	96 (31.8)	135 (44.7)	71 (23.5)
9. When working, do you wear an apron?	62 (20.5)	121 (40.1)	119 (39.4)
10. When working, do you use a mask?	91 (30.1)	137 (45.4)	74 (24.5)
11. When working, do you wear a cap?	69 (22.8)	100 (33.1)	133 (44.0)
12. When working, do you wear gloves?	88 (29.1)	142 (47.0)	72 (23.8)
13. Do you use a tissue/cloth when coughing or sneezing?	77 (25.5)	92 (30.5)	133 (44.0)
14. Do you wash and sanitize the knife after chopping raw chicken or meat or other raw food?	85 (28.1)	155 (51.3)	62 (20.5)
15. Do you rub your hands on your face, hair, etc. while working?	134 (44.4)	133 (44.0)	35 (11.6)
16. Do you reuse the oil?	156 (51.7)	117 (38.7)	29 (9.6)
17. Do you smoke in your work place?	107 (35.4)	122 (40.4)	73 (24.2)
Food safety practices score, *n* (%)	Poor	Moderate	Good
	144 (47.7)	144 (47.7)	14 (4.6)

*Note*: Mean food safety practices score: Mean = 17.78 (SD = 5.74, range = 1–34).

**TABLE 5:** Association of vendors’ socio‐demography with food safety knowledge, attitudes and practices in Chattogram city, Bangladesh (*n* = 302)

	Knowledge score			Attitudes score			Practices score		
Characteristics	Mean	SD	*P*	Mean	SD	*P*	Mean	SD	*P*
**Gender** **^a^**									
Male	9.11	4.11	0.330	8.52	3.38	0.570	17.70	5.99	0.614
Female	8.55	4.36		8.25	3.94		18.05	4.78	
**Age (years)** ^b^									
<25	6.77	4.48	**≤0.001**	6.40	4.11	**≤0.001**	18.08	6.96	0.475
25–50	9.36	4.14		8.70	3.28		17.58	5.59	
> 50	9.92	1.85		10.19	2.56		18.96	4.33	
**Experience of work (years)** ^b^									
<5	7.70	4.83	**0.012**	7.58	3.75	0.051	19.11	6.34	**0.027**
5–9	9.52	3.91		8.66	3.37		17.53	5.66	
10–20	9.40	3.57		9.12	3.18		17.29	4.75	
>20	8.22	4.11		8.00					
**Educational level** ^b^									
No Formal Education	8.30	4.30	0.116	7.75	3.97	**0.002**	16.56	5.36	**≤0.001**
Primary	9.27	4.03		8.54	3.13		17.47	5.06	
Secondary	9.34	3.83		9.05	2.78		19.20	6.02	
Higher Secondary	10.00	6.02		10.85	3.62		26.14	7.55	
Under Graduation	12.20	3.70		12.60	3.97		25.60	7.09	
**Marital status** ^b^									
Single	7.51	4.30	**≤0.001**	7.11	3.80	**≤0.001**	17.28	6.32	0.152
Married	9.81	3.99		9.10	3.07		18.32	5.37	
Divorced or Separated	10.59	2.01		10.68	2.45		16.31	4.86	
**Household income (BDT)** ^b^									
<5000	4.36	3.20	**≤0.001**	4.73	2.40	**≤0.001**	16.21	4.02	0.054
5000–10000	7.76	4.25		7.44	3.88		16.86	6.08	
10001–20000	10.19	3.75		9.19	2.98		18.85	5.87	
>20000	10.23	3.52		9.74	2.78		18.10	5.29	
**Residence** ^b^									
City area	9.787	4.02	**≤0.001**	8.67	3.58	0.156	17.62	5.72	0.714
Sur Urban	7.98	4.09		8.52	2.97		17.78	5.47	
Rural Area	7.09	4.02		7.60	3.72		18.37	6.15	
**Food safety training** ^a^									
Yes	5.31	1.68	0.279	8.40	4.37	0.954	21.20	8.62	0.056
No	8.94	0.24		8.46	3.48		17.66	5.60	

*Note*: Bold values indicates statistically significant (*p* < 0.05).

^a^ *P*‐value was determined by independent sample *t*‐test.

^b^ *P*‐value was determined by one‐way ANOVA.

Table 6 demonstrates the correlation of food safety knowledge, attitude and practice scores with some selected explanatory variables. The vendors’ age (*r* = +0.254), education (*r* = +0.142), work experience (*r* = +0.133) and household income (*r* = +0.347) were positively correlated with their food safety knowledge. Food safety attitude had a significant positive correlation with age (*r* = +0.267), educational level (*r* = +0.222), work experience (*r* = +0.163), household income (*r* = +0.349) and knowledge of food safety (*r* = +0.645). Safety practices were positively correlated with education (*r* = +0.293), household income (*r* = +0.123) knowledge (*r* = +0.218) and attitudes (*r* = +0.363), although negatively correlated with age (*r* = ‐0.077) and work experience (*r* = ‐0.124).

**TABLE 6:** Correlation between food safety knowledge, attitudes, and practices scores and selected independent variables

**Variables**	1	2	3	4	5	6	7
1. Age^a^	–						
2. Education^a^	‐0.077 (0.184)	–					
3. Work experience^a^	0.714 (≤0.01)	‐0.107 (0.604)	–				
4. Household income^a^	0.474 (≤0.01)	0.094 (0.102)	0.323 (≤0.01)	–			
5. Knowledge^b^	0.254 (≤0.01)	0.142 (0.013)	0.133 (.021)	0.347 (≤0.01)	–		
6. Attitudes^b^	0.267 (≤0.01)	0.222 (≤0.01)	0.163 (.004)	0.349 (≤0.01)	0.645 (≤0.01)	–	
7. Practice^b^	‐0.077 (0.185)	0.293 (≤0.01)	‐0.124 (.031)	0.123 (.033)	0.218 (≤0.01)	0.363 (≤0.01)	–

*Note*: Correlation is significant at the 0.01 level (2‐tailed). *P*‐value in the parenthesis.

^a^ Spearman Rank‐order correlation.

^b^ Pearson correlation.

## DISCUSSION

In developing countries, street food business provides low‐cost ready‐to‐eat meals for the consumers and create an informal sector of the economy for marginal people. However, unsafe food preparation and supply by street‐food vendors often made a food safety threat to public health. Maintaining the quality and safety of street food is associated with the street food vendors’ appropriate food handling practices to reduce foodborne illness. Thus, the current study explored street‐food vendors’ food safety knowledge, attitudes and practices in Chattogram city, Bangladesh.

The participants presented a lack of knowledge of foodborne pathogens, as more than three‐fourths could not identify the pathogens*, Salmonella* and *Staphylococcus*. These findings were echoed by previous research conducted in China [35], Vietnam [36] and Haiti [1], where over 90% of vendors had no knowledge of it. However, knowledge of transmission of bloody diarrhea (67.5%) and typhoid fever (62.3%) by food was high which is similar to a previous study in Haiti [1], signifying participants have some understanding of foodborne diseases. The street‐food vendors were also knowledgeable that washing hands before work (71.5%) and using gloves whilst handling foods (68.2%) reduces the risk of contamination. These results were in‐line with previous studies of street food vendors conducted in China [35], Vietnam [36] and Haiti [1]. Having adequate knowledge of foodborne diseases (such as diarrhea and typhoid fever) and food safety precaution measures (such as, hand washing, using gloves, etc.) does not guarantee the vendors’ food safety practices. This can be exemplified by this study's finding that the majority of vendors either did not know or wrongly knew the importance of taking leave from work when they experienced an infectious skin disease.

The street food vendors’ low knowledge of foodborne pathogens in this study is consistent with previous studies, conducted in Ethiopia [29], Malaysia [28] and Iran [38] that reported poor knowledge of food hygiene among street food vendors. Evidence from the literatures [39, 40, 41] indicates that food safety information is important for street food vendors, and training programs on food safety are recommended to help them translate their knowledge into hygienic practices.

With regards to the factors associated with food safety knowledge, our findings revealed that vendors’ age, educational level, place of residence, income level, work experience and marital status were significantly associated with food safety knowledge. Similar to our findings, Ma et al. showed a significant association between age and knowledge of food safety [35]. Relatedly, previous studies conducted in the South‐western region of Bangladesh [19] and Handan, China [35] reported a significant influence of the street food vendors’ education on their knowledge of food safety. This has been elaborated by Hossen et al. [19] who reported that street food vendors with secondary and tertiary education had good food safety knowledge. Such finding is not surprising as educated people are more likely exposed to food hygiene and precaution training that may help them to comprehend their learnings on food safety.

In contradiction to our finding, Ma et al. [35] showed no significant association of work experience with street food vendors’ food safety knowledge. Ma et al. [35] specified that knowledge of food safety was significantly associated with street food vendors’ place of residence, suggesting that vendors living in rural areas may lack food safety knowledge. The rurality in Bangladesh, and in other countries has been characterized by people with a lower educational level and poor income. Past studies [42, 43] also associated work experience with street food vendors’ food safety knowledge. Holding all things constant, those who have been street food handlers for a longer period are more likely to learn from experience and any tailored educational interventions compared to those who have few years of working experience.

The current study found participants’ household income was significantly associated with street food vendors’ food safety knowledge and attitudes. A recent study showed that food handlers with higher income were more likely to have higher food safety knowledge, and this was explained by the notion that financial solvency might allow them to afford professional training on food safety, which in turn translates to higher food safety knowledge and attitudes [26]. In Bangladesh, many street food vendors are constrained by the poor socioeconomic conditions in their families [5]. Thus, financial assistance (such as microcredit, governmental or non‐governmental financial aids, etc.) improves street food vendors’ quality of life as well as helps to maintain proper sanitary and hygiene indicators throughout the infrastructure.

With regard to marital status, previous studies [44, 45] have also found a statistically significant association between marital status and food safety knowledge with those not married having a high knowledge score. This is incongruent with the findings in the current study as those who were divorced or separated had higher food safety knowledge scores. A qualitative study is warranted to unravel such a finding.

Bou‐Mitri et al. and Mukherjee et al. identified vendors’ attitude as critical factors that has an influence on their food safety practice and lessen the recurrence of foodborne disease [46, 47]. Our study showed a negative attitude toward food safety among the majority of the street‐food vendors located in Chattogram city, Bangladesh. This finding contradicts the finding of Mukherjee et al. [47] and Tuglo et al. [48] who reported a favourable attitude among the vendors in India as far as the food safety issues are concerned. However, this finding corresponds with a study conducted in Malaysia [28]. The plausible reason could be due to the differences in study settings and socio‐demographic characteristics. It was found that food safety attitude had a significant positive correlation with age, educational level, marital status and knowledge of food safety which has also been reported in previous studies [23, 49]. The negative attitude found in this current study calls for the need to ensure that there is behavioural change communication (BCC) with emphasis on motivation and training for the food handlers [49].

According to Mukherjee et al. [47], practice is the way in which people demonstrate their knowledge and attitude through their actions. Our study reported poor to moderate levels of food safety practices among street‐food vendors, and this can be related to their low food safety knowledge. Our finding contradicts that of previous studies conducted in Ethiopia [50], Romania [22] and Saudi Arabia [24]. This implies that food safety knowledge and attitudes are crucial elements in the promotion of good food safety practices. To improve food safety practices, it is imperative to intensify education for food handlers to gain more positive attitudes towards food safety.

### Strengths and limitations of the study

This study has several strengths. Our study is one of the first to explore factors affecting street food vendors’ food safety knowledge, attitudes and practices in the second most densely populated city in Bangladesh (Chattogram), where food safety of street foods remains a public health concern. The findings of this study provide baseline information for policymakers and public health practitioners and may inform the Bangladesh Food Safety Authority (BFSA) about where and how to intervene in policies and strategies in improving food safety in this region. Analytical rigor and detailed methodology are other strengths of this study. However, the study is limited by the cross‐sectional design which limits causal inference. Since this study investigated a specific area of Bangladesh (i.e., Chattogram city), findings cannot be generalized to other regions (e.g., hill tracts regions, island areas, and rural areas) of the country. Face‐to‐face interviews, which took into account street food vendors' attitudes and self‐reported practices, could have contributed to the occurrence of social desirability and information bias.

## CONCLUSIONS

Generally, Bangladeshi street food vendors in our study were found to have low food safety knowledge, negative levels of food safety attitudes, and poor to moderate levels of food safety practices. Several sociodemographic factors are associated with street food vendors’ food safety knowledge (age, marital status, income, residence and work experience), attitudes (age, marital status, income and education level) and practices (education level and work experience). Given the associated public health concerns that can result from inadequate or a lack of food safety including poor food handling or preparation practices, there is a need for targeted strategies and interventions programs such as, educating about foodborne pathogens and its adverse impact, food safety trainings, awareness campaigns and financial support to improve street food vendors’ knowledge, attitudes and practices of food safety. BFSA along with respective local authorities should take concrete efforts to ensure the safety of street foods and improve the knowledge, attitudes and level of practices among street food vendors through targeted informational campaigns and strict monitoring and regulation of street food vendors to prevent foodborne illness and related outcomes. To get healthy SVF in Bangladesh, some recommendations are: (i) to institutionalize healthy street food vending system, (ii) to provision of laws and regulations for SVFs as an integral part of urban development, (iii) to provide financial, health and social welfare support to the street food vendors and (iv) to formulate street food shop operating conditions and facilities (such as proper hygiene and sanitary indicator, potable water, proper waste management, etc.). Further qualitative and longitudinal studies are warranted to acquire more detailed information about street food vendors’ knowledge, attitudes and practice, such as social and psychological factors that influence their attitudes and practices, in order to drive food safety education.

## CONFLICT OF INTEREST

The author(s) declare(s) that there is no conflict of interest regarding the publication of this article. This research did not receive any specific grant from funding agencies in the public, commercial, or not‐for‐profit sectors.

## AUTHOR CONTRIBUTIONS

Mohammad Tazrian Abid: Study design, writing‐ original draft & formal analysis. Md. Hasan Al Banna: Conceptualization, study design, writing‐ original draft preparation, critical reviewing & editing. Mohammad Hamiduzzaman: Visualization, validation & writing‐ critical reviewing and editing. Abdul‐Aziz Seidu: Validation, writing‐ original draft preparation. Satyajit Kundu, Humayra Rezyona, Tasnim Rahman Disu: Visualization, validation & writing‐ reviewing and editing. Nargees Akter & Md Khaleduzzaman: Data curation, writing‐ original draft preparation. Bright Opoku Ahinkorah: Validation, writing‐ original draft preparation. Md Shafiqul Islam Khan: Conceptualization, writing‐ reviewing and editing & supervision. All authors read and approved the final version of the manuscript.

## ETHICS STATEMENT

All study protocols and procedures were reviewed and approved by the department of the principal author (approval number: FMB:15/12/2020:05). Written consent was obtained from the participants (street food vendors) after discussing the purpose of the study, confidentiality of their data, and after assuring the participant that this research would not be harmful to them.

## Data Availability

The datasets of the current study are available from the corresponding author on reasonable request.
